# Anti-CD20–atezolizumab–polatuzumab vedotin in relapsed/refractory follicular and diffuse large B-cell lymphoma

**DOI:** 10.1007/s00432-021-03847-5

**Published:** 2022-02-18

**Authors:** Max S. Topp, Herbert Eradat, Axel Florschütz, Andreas Hochhaus, Tomasz Wrobel, Jan Walewski, Wanda Knopinska-Posluszny, Abraham S. Kanate, Ewa Lech-Maranda, Uta Brunnberg, Surya Chitra, Tina G. Nielsen, Gila Sellam, Mahesh Shivhare, Izidore S. Lossos

**Affiliations:** 1 Medizinische Klinik und Poliklinik II, Universitätsklinikum Würzburg, Anstalt des öffentlichen Rechts, Josef-Schneider-Straße 2, 97080 Würzburg, Germany; 2 Division of Hematology-Oncology, David Geffen School of Medicine at UCLA, Los Angeles, CA USA; 3 Städtisches Klinikum Dessau, Dessau-Roβlau, Germany; 4 Klinik für Innere Medizin II, Universitätsklinikum Jena, Jena, Germany; 5 Department of Hematology, Wrocław Medical University, Wrocław, Poland; 6 Narodowy Instytut Onkologii im, Marii Skłodowskiej-Curie - Panstwowy Instytut Badawczy, Warsaw, Poland; 7 Uniwersytet Warmińsko-Mazurski, Olsztynie, Poland; 8 West Virginia University Cancer Institute, Morgantown, WV USA; 9 Institute of Hematology and Transfusion Medicine, Warsaw, Poland; 10 Universitätsklinikum Frankfurt, Frankfurt, Germany; 11 Genentech, Inc., South San Francisco, CA USA; 12 F. Hoffmann-La Roche Ltd, Basel, Switzerland; 13 Roche Products Limited, Welwyn Garden City, UK; 14 Sylvester Comprehensive Cancer Center, University of Miami, Miami, FL USA

**Keywords:** B-cell non-Hodgkin lymphoma, Diffuse large B-cell lymphoma, Follicular lymphoma, Immunotherapy, Relapsed/refractory

## Abstract

**Purpose:**

New therapies are needed for relapsed/refractory (R/R) B-cell non-Hodgkin lymphoma. This phase 1b, open-label trial evaluated two anti-CD20-based triplet combinations.

**Methods:**

Patients with R/R follicular lymphoma (FL; *n* = 13) were treated with obinutuzumab, atezolizumab, and polatuzumab vedotin (G-atezo-pola; 1.4 mg/kg/1.8 mg/kg) and patients with R/R diffuse large B-cell lymphoma (DLBCL; *n* = 23) received rituximab (R)-atezo-pola. The primary efficacy endpoint was complete response (CR) at end of induction (EOI) by PET-CT (investigator assessed; modified Lugano 2014 criteria). Safety endpoints were also assessed.

**Results:**

13 FL patients were treated and evaluable for safety; 2/23 DLBCL patients did not receive treatment and were not included in the safety population. Median observation time was 23.3 and 5.7 months in the FL and DLBCL cohorts, respectively. At EOI, CR rates in FL patients treated with G-atezo-pola at pola doses of 1.4 mg/kg (*N* = 3) and 1.8 mg/kg (*N* = 7) were 33% and 14%, respectively. In DLBCL patients receiving R-atezo-pola, the CR rate at EOI was 13%. In the FL cohort, 62% of patients experienced a grade 3–5 adverse event (AE; including two deaths) and 31% developed a serious AE (SAE). In DLBCL patients, R-atezo-pola was associated with a lower incidence of grade 3–5 AEs (24%; one death) and SAEs (10%). In both cohorts, the most common grade 3–5 AEs were hematologic toxicities.

**Conclusion:**

Based on these safety issues, considered as related specifically to G-atezo-pola, and limited efficacy, no further development of either combination is planned.

**Trial registration:**

NCT02729896; Date of registration: April 6, 2016.

**Supplementary Information:**

The online version contains supplementary material available at 10.1007/s00432-021-03847-5.

## Introduction

Immunotherapy is an emerging approach for the treatment of relapsed/refractory (R/R) B-cell non-Hodgkin lymphoma, for which, there is an unmet need for new therapies, including the use of anti-programmed death-1 (anti-PD-1) therapy. Previous studies in B-cell malignancies have demonstrated the potential of combining atezolizumab (anti-PD-ligand 1 [anti-PD-L1] antibody) (US FDA 2019; TECENTRIQ^®^ 2019) or polatuzumab vedotin (pola; anti-CD79b antibody-drug conjugate) (US FDA 2019) with anti-CD20-based therapies, with encouraging response rates (US FDA 2019; TECENTRIQ^®^ 2019; Morschhauser et al. 2019; Phillips et al. 2016; Palomba et al. 2017). Data from the phase 1b/2 GO29365 study supported Food and Drug Administration (FDA)-accelerated approval of the first triplet combination therapy in R/R diffuse large B-cell lymphoma (DLBCL), incorporating the anti-CD20 antibody, rituximab (R), with pola and bendamustine. Two phase 3 trials, AUGMENT and MAGNIFY, provided evidence for FDA approval of lenalidomide in combination with R for R/R follicular lymphoma (FL) and marginal zone lymphoma. The proven efficacy of anti-CD20 antibody-based therapy supports further investigation into new combinations (Sehn et al. 2016; Ren et al. 2015; Gao et al. 2010). We present the primary analysis of a phase 1b, open-label multicenter trial of two anti-CD20-based triplet combinations in R/R FL and DLBCL (BO29561; NCT02729896), where recruitment was stopped due to safety concerns. Patients with R/R FL were treated with obinutuzumab (G), atezolizumab (atezo), and pola (G-atezo-pola); patients with R/R DLBCL received R-atezo-pola.

## Methods

### Patients, treatment and endpoints

Eligible patients had previously received ≥ 1 prior chemoimmunotherapy regimen, including an anti-CD20 antibody (see Online Supplemental Data for eligibility criteria). FL patients were first enrolled in a dose-escalation phase to establish the recommended phase 2 dose (RP2D) of pola within the G-atezo-pola regimen. During dose escalation, FL patients received up to six 21-day cycles of obinutuzumab (1000 mg intravenously [IV], Day [D]1, D8, D15 of Cycle [C]1, and D1 of C2‒6) and atezolizumab (1200 mg IV, D1 of C2–6) plus pola (1.4 or 1.8 mg/kg IV, D1 of C1–6). Subsequently, patients entered an expansion phase and received obinutuzumab and atezolizumab (same doses) plus pola at the RP2D (1.8 mg/kg). Patients who achieved at least stable disease at the end of induction (EOI; 6–8 weeks after D1C6) proceeded to obinutuzumab maintenance (1000 mg every 2 months) and atezolizumab (840 mg, D1 and D2 every month) for up to 2 years, or until progressive disease (PD). Unlike the FL cohort, the first seven DLBCL patients entered a safety run-in; once safety criteria were met, the cohort was expanded. All DLBCL patients received up to six 21-day cycles of rituximab (375 mg/m^2^ IV, D1 of C1–6), atezolizumab (1200 mg, D1 of C2–6), and pola (1.8 mg/kg, D1 of C1–6). Patients with at least a partial response (PR) at EOI (6–8 weeks after D1 of C6) received rituximab consolidation (375 mg/m^2^, D1 every 2 months) and atezolizumab (840 mg, D1 and D2 every month) for up to 8 months, or until PD.

The primary efficacy endpoint was complete response (CR) at EOI by PET-CT (investigator assessed; modified Lugano 2014 criteria) (Cheson et al 2016a, b). Modifications to the standard Lugano criteria were as follows: for the designation of a PR on PET, criteria for CR or PR on CT scan had to be met; if bone marrow (BM) involvement was present at baseline, CR had to be confirmed with a negative BM result at end of induction (Cheson et al. 2016a, b).

Overall response rate (CR + PR) by PET-CT was a secondary endpoint. Safety endpoints were the RP2D of pola in combination with G-atezo in FL patients (based on dose-limiting toxicities in C1 and C2), and the safety/tolerability of the two regimens in each cohort. The study was conducted in accordance with the Declaration of Helsinki, Good Clinical Practice guidelines, and applicable local laws and regulations. Study materials, including the protocol, were approved by the institutional review boards/ethics committees at participating centers and patients provided written informed consent.

### Statistical analysis

Safety analyses were conducted in all patients who received ≥ 1 dose of any study drug (safety population) and are presented by treatment cohort using descriptive statistics. All safety analyses include data collected up to the clinical cut-off date of September 4, 2018.

Efficacy analyses were undertaken in all patients who received ≥ 1 dose of each component of the G-atezo-pola or R-atezo-pola combinations (efficacy population). Patients with FL who received polatuzumab vedotin at the RP2D during the dose-escalation phase of the study were pooled for analysis with FL patients who were treated in the expansion phase. Similarly, DLBCL patients from the safety run-in were pooled for analysis with DLBCL patients treated in the expansion phase. Due to early discontinuation of atezolizumab for all patients on March 1, 2018, the efficacy population included patients who were not exposed or only partially exposed to atezolizumab. To evaluate the efficacy of induction treatment (primary objective), censoring was applied to the efficacy analyses from March 1, 2018. Response rates are presented using descriptive statistics.

## Results

**Fig. 1:**
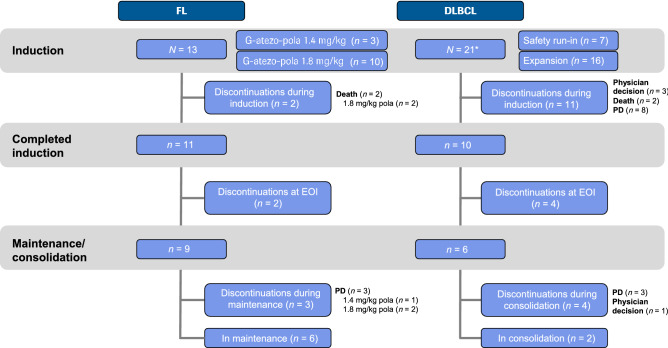
Patient deposition. *Two additional patients with DLBCL were enrolled but did not receive study treatment and were not included in the safety or efficacy populations. *Atezo* atezolizumab, *DLBCL* diffuse large B-cell lymphoma, *EOI* end of induction, *FL* follicular lymphoma, *G* obinutuzumab, *PD* progression of disease, *pola* polatuzumab vedotin, *R* rituximab, *R/R* relapsed refractory

Between November 9, 2016 and March 1, 2018, 36 patients (R/R FL, *n* = 13; R/R DLBCL, *n* = 23) were enrolled. The clinical cut-off for safety was September 4, 2018. All 13 FL patients were treated and evaluable for safety; 2/23 DLBCL patients did not receive treatment and were not included in the safety population. At the time of data censoring (March 1, 2018), 9/10 FL patients and 10/16 DLBCL patients were evaluable for response. Patient disposition (Fig. 1) and baseline characteristics (Table 1) are presented. Median observation time was 23.3 months (range 3.1–23.7) and 5.7 months (range 0.9–15.4) in the FL and DLBCL cohorts, respectively. On March 1, 2018 (after an interim analysis), study recruitment was stopped permanently due to unexpected safety signals related specifically to G-atezo-pola, including two fatal adverse events (AEs) (Topp et al. 2020) and limited efficacy. Atezolizumab was discontinued in both cohorts, and due to the lack of evidence supporting extended rituximab monotherapy in DLBCL, rituximab consolidation was ceased in the DLBCL cohort; however, the study remains active for enrolled patients.

**Table 1:** Demographics and baseline characteristics of all R/R FL and DLBCL patients (safety population)

	R/R FL G-atezo-pola		R/R DLBCL R-atezo-pola
	Pola 1.4 mg/kg (*N* = 3)	Pola 1.8 mg/kg (*N* = 10)	Pola 1.8 mg/kg (*N* = 21)
Median age, years (range)	54 (38–65)	58.5 (31–71)	68 (21–92)
Male, *n* (%)	3 (100)	6 (60)	13 (62)
Ann Arbor stage at diagnosis (III–IV), *n* (%)	2 (67)	9 (90)	18 (86)
FLIPI score, *n* (%)			
0–1 (low risk)	2 (67)	3 (30)	–
2 (intermediate risk)	1 (33)	1 (10)	–
3–5 (high risk)	0	6 (60)	–
IPI score, *n* (%)			
0–2 (low risk)	–	–	7 (33)
3–5 (intermediate-to-high risk)	–	–	14 (67)
ECOG PS 0–1, *n* (%)	3 (100)	10 (100)	18 (86)
Bulky disease (> 7.5 cm), *n* (%)	0	6 (60)	6 (29)
Bone marrow infiltration, *n* (%)	0	2 (20)	4 (20)
Extranodal involvement, *n* (%)	1 (33)	7 (70)	16 (76)
Elevated LDH, *n* (%)	0	2 (20)	15 (71)
Refractory to last line, *n* (%)	1 (33)	8 (80)	20 (95)
Relapsed on last line, *n* (%)	2 (67)	2 (20)	1 (5)
Median prior lines of therapy (range)	2 (1–5)	2 (1–4)	2 (1–8)
Prior stem cell transplant, *n* (%)	0	1 (10)	6 (29)

*Atezo* atezolizumab, *DLBCL* diffuse large B-cell lymphoma, *ECOG PS* Eastern Cooperative Oncology Group performance status, *FL* follicular lymphoma, *FLIPI* Follicular Lymphoma International Prognostic Index, *G* obinutuzumab, *IPI* International Prognostic Index, *LDH* lactate dehydrogenase, *pola* polatuzumab vedotin, *R* rituximab, *R/R* relapsed/refractory

At EOI, CR rates in FL patients treated with G-atezo-pola at pola doses of 1.4 mg/kg (*N* = 3) and 1.8 mg/kg (*N* = 7) were 33% (overall response rate [ORR], 33%) and 14% (ORR, 57%), respectively (Table 2). In DLBCL patients who received R-atezo-pola, the CR rate at EOI was 13% (ORR, 25%).

**Table 2:** Response at end of induction (investigator assessed^a^) in patients with R/R FL and DLBCL

*n* (%)	R/R FL G-atezo-pola^b^		R/R DLBCL R-atezo-pola
	Pola 1.4 mg/kg (*N* = 3)	Pola 1.8 mg/kg (*N* = 7)	Pola 1.8 mg/kg (*N* = 16)^c^
ORR	1 (33)	4 (57)	4 (25)
CR	1 (33)	1 (14)	2 (13)
PR	0	3 (43)	2 (13)
SD	1 (33)	1 (14)	1 (6)
PD	1 (33)	1 (14)	5 (31)
Not evaluable	0	1 (14)	6 (38)

Only patients who received the last dose of study drug in induction before March 1, 2018 or patients with PD or death before March 1, 2018 are included

*Atezo* atezolizumab, *CR* complete response, *DoR* duration of response, *DLBCL* diffuse large B-cell lymphoma, *EOI* end of induction, *FL* follicular lymphoma, *G* obinutuzumab, *ORR* overall response rate, *PD* progression of disease, *pola* polatuzumab vedotin, *PR* partial response, *R* rituximab, *R/R* relapsed refractory, *SD* stable disease

^a^Assessed by positron emission tomography-computed tomography according to modified Lugano 2014 response criteria

^b^To date, a CR has been maintained from EOI in two patients receiving G-atezo-pola; DoR, 21.42 months and 2.79 months, respectively

^c^To date, a CR has been maintained from EOI in two patients receiving R-atezo-pola; DoR, 10.84 months and 8.97 months, respectively

Safety assessment in FL patients established the RP2D of pola in the G-atezo-pola regimen as 1.8 mg/kg, based on an absence of C1/C2 dose-limiting toxicities in three patients treated with the 1.4 mg/kg dose. This is the dose approved for use in combination with rituximab and bendamustine for R/R DLBCL (US FDA 2019). In the FL cohort, 8/13 patients (62%) experienced a grade 3–5 AE (including two deaths) and 4/13 patients (31%) developed a serious AE (SAE) (Table 3). One out of 13 patients (8%) experienced pneumonitis (grade 3) and 3/13 patients (23%) experienced peripheral neuropathy (unresolved in one patient at the time of analysis/death). In DLBCL patients, R-atezo-pola was associated with a lower incidence of grade 3–5 AEs (5/21 patients [24%]; one death) and SAEs (2/21 patients [10%]) (Table 3). One out of 21 patients (5%) experienced peripheral neuropathy (unresolved at the time of analysis) and no events of pneumonitis were reported in the DLBCL cohort. Across both cohorts, the most common grade 3–5 AEs were hematologic toxicities, although most events were isolated and reported in one patient only (Table 4).

**Table 3:** Summary of adverse events reported in all R/R FL and DLBCL patients (safety population)

	R/R FL G-atezo-pola (*N* = 13)	R/R DLBCL R-atezo-pola (*N* = 21)
Patients with ≥ 1 AE, *n* (%)	13 (100.0)	17 (81.0)
Grade 3–5 AE, *n* (%)	8 (62.0)	5 (24.0)
Grade 5 (fatal) AE, *n* (%)	2 (15.0)^a^	1 (5.0)^b^
Serious AE, *n* (%)	4 (31.0)	2 (10.0)
AE leading to any drug discontinuation, *n* (%)	2 (15.0)	–
AE leading to pola dose reduction, *n* (%)	1 (8.0)^c^	–
AE leading to any dose interruption or modification, *n* (%)	6 (46.0)	3 (14.0)

*AE* adverse event, *atezo* atezolizumab, *DLBCL* diffuse large B-cell lymphoma, *FL* follicular lymphoma, *G* obinutuzumab, *pola* polatuzumab vedotin, *R* rituximab, *R/R* relapsed/refractory

^a^Bronchopulmonary aspergillosis (*n* = 1) and unknown (*n* = 1)

^b^Pleural effusion (*n* = 1; a complication of sepsis)

^c^Peripheral neuropathy (*n* = 1)

**Table 4:** Grade 3–5 adverse events reported in ≥ 2 R/R FL and DLBCL patients

Preferred term, *n* (%)	R/R FL G-atezo-pola (*N* = 13)^a^		R/R DLBCL R-atezo-pola (*N* = 21)^b^
	Pola 1.4 mg (*N* = 3)	Pola 1.8 mg (*N* = 10)	Pola 1.8 mg (*N* = 21)
Neutropenia	1 (33)	2 (20)	2 (10)
Febrile neutropenia	–	2 (15)^c^	0
Thrombocytopenia	–	3 (23)	0
Leukopenia	–	2 (15)	1 (5)
Anemia	–	1 (8)^c^	1 (5)
Stomatitis	–	2 (15)^c^	0

*Atezo* atezolizumab, *DLBCL* diffuse large B-cell lymphoma, *FL* follicular lymphoma, *G* obinutuzumab, *pola* polatuzumab vedotin, *R* rituximab, *R/R* relapsed/refractory, *SAE* serious adverse event

^a^Additional isolated grade 3–5 events in patients with R/R FL were bronchopulmonary aspergillosis (SAE; grade 5), pelvic abscess, pneumonitis (SAE; grade 3; resolving), pulmonary embolism, pyrexia (SAE; grade 3; resolved), hypokalemia, sacroiliitis, Guillain-Barré syndrome (suspected; SAE; grade 3; resolving), dermatitis (SAE; grade 3; not resolved), increased lipase, increased gamma-glutamyl transferase, increased C-reactive protein (SAE; grade 3; resolved), increased white blood cell count, increased alanine aminotransferase, and unknown death (SAE; grade 5)

^b^Additional isolated grade 3–5 events in patients with R/R DLBCL were decreased appetite, diabetes mellitus, abdominal pain (SAE; grade 3; not resolved), fatigue, pneumonia, sepsis (SAE; grade 4; resolved), lethargy (SAE; grade 3; resolved), depression, acute respiratory failure, dyspnea, and pleural effusion (SAE; grade 5)

^c^One event was classified as an SAE (grade 3; resolving)

Both FL patients who died from AEs experienced a new constellation of immune toxicities, which manifested as concomitant severe cutaneous toxicity (dermatitis/erythema), stomatitis, and ocular AEs (scleritis/conjunctivitis) refractory to standard corticosteroid and other immunosuppressive treatment; and resembled an autoimmune disease (Stevens-Johnson syndrome) resulting in treatment discontinuation (Topp et al. 2020; Online Supplemental Figure S1). In both cases, patients developed opportunistic infections and other complications related to immunosuppressive therapy. The ultimate cause of death was bronchopulmonary aspergillosis and unknown (no autopsy was performed), respectively. These toxicities were considered by investigators as related specifically to G-atezo-pola. Such presentations were not seen following treatment of DLBCL patients with R-atezo-pola.

## Discussion

Despite prior studies demonstrating the potential of anti-CD20 doublets with atezolizumab or pola in R/R non-Hodgkin lymphoma (Morschhauser et al. 2019; Phillips et al. 2016; Palomba et al. 2017) response rates did not appear to improve further in FL and DLBCL patients treated with G-atezo-pola and R-atezo-pola, respectively. In the phase 2 ROMULUS study, CR rates in R/R DLBCL and R/R FL patients treated with R-pola were 16% (ORR 45%) and 29% (ORR 57%), respectively (Morschhauser et al. 2019). Comparable CR rates were also reported in a phase 1b/2 study investigating G-pola (R/R DLBCL: 28.5% [ORR 52%]; R/R FL: 30% [ORR 78%]) (Phillips et al. 2016).

In the FL cohort, more patients experienced grade 3–5 AEs with G-atezo-pola compared with patients with DLBCL receiving R-atezo-pola. The potentially better tolerability with R-atezo-pola in patients with DLBCL versus G-atezo-pola in patients with FL may be due to differences between the anti-CD20 drugs; rituximab may cause less profound B-cell depletion than obinutuzumab (Freeman and Sehn 2018) and, therefore, fewer immune-related AEs (Liudahl and Coussens 2018) or a lower exposure to atezolizumab and shorter observation time in the DLBCL cohort.

The toxicities that led to fatal outcomes in two patients with FL were considered by investigators as related specifically to G-atezo-pola; however, cutaneous, mucosal, and ocular immune toxicities are already known class-risks for anti-PD-1/PD-L1 therapies, such as atezolizumab (Michot et al. 2016; Brahmer et al. 2018; Collins et al. 2017). The authors of the present study hypothesized potential underlying mechanisms that led to the fatal outcome of these two patients. They suggested that the incidence and severity of these events, known to be associated with checkpoint inhibitors, may be exacerbated in the context of a profound dysregulation of the immune system: obinutuzumab- and polatuzumab vedotin-mediated B-cell suppression, and in particular, regulatory B-cell depletion (Kumar et al. 2015; Goldinger et al. 2016; Zamani et al. 2016; Dai et al. 2014). Furthermore, both patients had received prior treatment with bendamustine, which has been reported to induce regulatory T-cell depletion (Cheson et al. 2016a, b), potentially contributing to the immune dysregulation. There is insufficient evidence to support an auristatin-derived direct toxicity.

Based on these safety issues, considered as related specifically to the G-atezo-pola triplet, and limited efficacy, no further development of either triplet combination is planned.

## Supplementary Information

Below is the link to the electronic supplementary material.

Supplementary file1 (DOCX 337 KB)

## Declarations

### Data availability

Qualified researchers may request access to individual patient level data through the clinical study data request platform (https://vivli.org/). Further details on Roche's criteria for eligible studies are available here: (https://vivli.org/members/ourmembers/). For further details on Roche's Global Policy on the Sharing of Clinical Information and how to request access to related clinical study documents, see here: (https://www.roche.com/research_and_development/who_we_are_how_we_work/clinical_trials/our_commitment_to_data_sharing.htm).
