# Supplementary material for: Anti-CD20–atezolizumab–polatuzumab vedotin in relapsed/refractory follicular and diffuse large B-cell lymphoma
Source: J Cancer Res Clin Oncol. 2022 Feb 18;149(2):811–7. doi: 10.1007/s00432-021-03847-5 (PMC9931830; doi:10.1007/s00432-021-03847-5)
Supplement: Supplementary file 1 — Supplementary file1 (DOCX 337 KB) [file 432_2021_3847_MOESM1_ESM.docx]

**Supplemental data**

**Journal of Cancer Research Clinical Oncology**

**Anti-CD20-atezolizumab-polatuzumab vedotin in relapsed/refractory follicular and diffuse large B-cell lymphoma**

**Authors**

Max S. Topp,^1^ Herbert Eradat,^2^ Axel Florschütz,^3^ Andreas Hochhaus,^4^ Tomasz Wrobel,^5^ Jan Walewski,^6^ Wanda Knopinska-Posluszny,^7^ Abraham S. Kanate,^8^ Ewa Lech-Maranda,^9^ Uta Brunnberg,^10^ Surya Chitra,^11^ Tina Nielsen,^12^ Gila Sellam,^12^ Mahesh Shivhare ^13^, Izidore S. Lossos^14^

**Affiliations**

^1^Medizinische Klinik und Poliklinik II, Universitätsklinikum Würzburg, Würzburg, Germany; ^2^Division of Hematology-Oncology, David Geffen School of Medicine at UCLA, Los Angeles, CA, United States; ^3^Städtisches Klinikum Dessau, Dessau-Roβlau, Germany; ^4^Klinik für Innere Medizin II, Universitätsklinikum Jena, Jena, Germany; ^5^Department of Hematology, Wrocław Medical University, Wrocław, Poland; ^6^Narodowy Instytut Onkologii im. Marii Skłodowskiej-Curie – Panstwowy Instytut Badawczy, Warsaw, Poland; ^7^Uniwersytet Warmińsko-Mazurski, Olsztynie, Poland; ^8^West Virginia University Cancer Institute, Morgantown, WV, United States; ^9^Institute of Hematology and Transfusion Medicine, Warsaw, Poland; ^10^Universitätsklinikum Frankfurt, Frankfurt, Germany; ^11^Genentech, Inc., South San Francisco, CA, United States; ^12^F. Hoffmann-La Roche Ltd, Basel, Switzerland; ^13^Roche Products Limited, Welwyn Garden City, United Kingdom; ^14^University of Miami, Sylvester Comprehensive Cancer Center, Miami, FL, United States.

**Correspondence:** Max S. Topp; [topp_m@ukw.de](mailto:topp_m@ukw.de)

**Inclusion criteria**

- Age ≥18 years.
- *Obinutuzumab, atezolizumab, and polatuzumab vedotin (G-atezo-pola) cohort:* relapsed/refractory (R/R) follicular lymphoma (FL) after treatment with ≥1 prior immunochemotherapy regimen that included an anti-CD20 monoclonal antibody and for which no other more appropriate treatment option exists (as determined by the investigator).
- *Rituximab, atezolizumab, and polatuzumab vedotin (R-atezo-pola) cohort*: R/R diffuse large B-cell lymphoma (DLBCL) after treatment with ≥1 prior immunochemotherapy regimen that included an anti-CD20 monoclonal antibody in patients not eligible for second-line combination (immuno)chemotherapy and autologous stem-cell transplantation (ASCT), or in those who have failed second-line combination (immuno)chemotherapy or have experienced disease progression following ASCT.
- Histologically documented, CD20-positive and fluorodeoxyglucose (FDG)-avid (i.e., positron emission tomography [PET]-positive lymphoma) with ≥1 bi-dimensionally measurable lesion.
- Eastern Cooperative Oncology Group (ECOG) performance status of 0-2.
- Availability of a representative tumor specimen and the corresponding pathology report for retrospective central confirmation of the diagnosis of FL or DLBCL.
- *For women who are not postmenopausal or surgically sterile:* agreement to remain abstinent or to use contraceptive methods that result in a failure rate of <1% per year during the treatment period for ≥5 months after the last dose of atezolizumab, ≥12 months after the last dose of rituximab, ≥12 months after the last dose of polatuzumab vedotin, and ≥18 months after the last dose of obinutuzumab.
- *For men*: agreement to remain abstinent or to use contraceptive measures that result in a failure rate of <1% per year during the treatment period and for ≥3 months after the last dose of obinutuzumab, rituximab, and atezolizumab, and for ≥5 months after the last dose of polatuzumab vedotin, and agreement to refrain from donating sperm during this same period.

**Exclusion criteria**

- Grade 3b FL.
- History of transformation of indolent disease to DLBCL.
- Known CD20-negative status at relapse or progression.
- Central nervous system lymphoma or leptomeningeal infiltration.
- Prior allogeneic stem cell transplantation or completion of ASCT within 100 days prior to Day 1 of Cycle 1 (D1C1).
- Prior anticancer therapy including: fludarabine or alemtuzumab within 12 months prior to D1C1; radioimmunoconjugate within 12 weeks prior to D1C1; monoclonal antibody or antibody drug conjugate (ADC) within five half-lives or 4 weeks prior to D1C1; radiotherapy, chemotherapy, hormonal therapy, or targeted small molecule therapy within 2 weeks prior to D1C1; or anti-programmed death-1 (anti-PD-1), anti-programmed death-ligand 1 (anti-PD-L1), anti-cytotoxic T-lymphocyte-associated protein 4 (CTLA4), anti-CD137/41-BB agonist, or anti-CD40 agonist antibodies.
- Treatment with systemic immunosuppressive medications, including, but not limited to, prednisone, azathioprine, methotrexate, thalidomide, or anti-tumor necrosis factor agents within 2 weeks prior to D1C1.
- History of solid organ transplantation.
- Severe allergic or anaphylactic reaction to humanized, chimeric, or murine monoclonal antibodies.
- Active infection.
- Testing positive for hepatitis B surface agent, total hepatitis B core antibody, or hepatitis C virus antibody at screening.
- Known history of human immunodeficiency virus (HIV)-positive status.
- Progressive multifocal leukoencephalopathy or autoimmune disease.
- Vaccination with a live virus vaccine or live attenuated vaccine within 28 days prior to D1C1.
- Pre-existing Grade >1 neuropathy.
- Major surgical procedure other than for diagnosis within 28 days prior to D1C1.
- Inadequate hematologic function, renal function, or liver function.
- Pregnant or lactating women.
- Life expectancy <3 months.

**Supplemental Figure S1.** Clinical features of oral mucosal involvement in the two patients with FL who died whilst experiencing drug-related toxicity


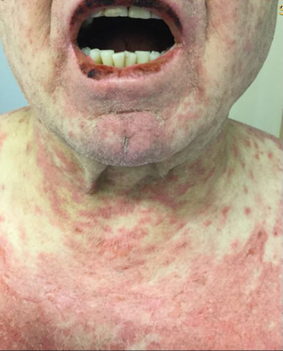
**
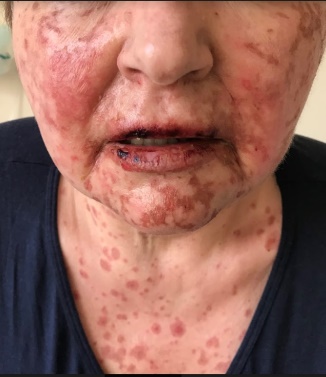
**

Clinical presentation of high-grade stomatitis with oral mucosal involvement in the two patients with FL who died while experiencing a constellation of drug-related immune-mediated adverse events after receiving treatment with G-atezo-pola.

Atezo, atezolizumab; FL, follicular lymphoma; G, obinutuzumab; pola, polatuzumab vedotin.

©Topp MS, Duell J, Abajo Guijarro AM, et al. Severe treatment-refractory T-cell-mediated immune skin toxicities observed with obinutuzumab/rituximab-atezo-pola in two patients with follicular lymphoma. Haematologica. 2020;105(5):e256–260.
